# The Interplay between Microbiota and Human Complex Traits

**DOI:** 10.3390/microorganisms11082066

**Published:** 2023-08-11

**Authors:** Laura Veschetti, Mirko Treccani, Giovanni Malerba

**Affiliations:** 1 GM Lab, Department of Neurosciences, Biomedicine and Movement Sciences, University of Verona, 37134 Verona, Italy; mirko.treccani@univr.it (M.T.); giovanni.malerba@univr.it (G.M.); 2 Infections and Cystic Fibrosis Unit, Division of Immunology, Transplantation and Infectious Diseases, IRCCS San Raffaele Scientific Institute, 20132 Milan, Italy

Microorganisms have been one of the most influential drivers propelling some of the greatest environmental and evolutionary changes in the landscape and biology of the entire planet. For example, the Great Oxidation Event (around 2.4–2.0 billion years ago) took place only thanks to the first carbon-fixing bacteria [1].

In the process of diversification and complexification, microorganisms established an ever-increasing number of connections by building biological networks and complex communities able to face even the most unfavorable conditions and to shape the features of their ecological niches. Interestingly, these interactions also have a crucial role for the life and health of multicellular organisms: they explicate key functions as aiding in the digestion and absorption of nutrients (e.g., short-chain fatty acid production and dietary fiber fermentation) [2], synthesizing vitamins (e.g., B1, B12, and K) [3], and helping to maintain a balanced immune system (e.g., pathogen exposure and hygiene hypothesis) [4].

A crawling web of interactions depicts the human–microorganism interplay as a dynamic equilibrium of connections opening and closing over time and space. A suggestive example is found in opportunistic pathogens, which are usually harmless, but easily determine diseases in immunocompromised and unhealthy individuals: *Legionella pneumophila* is ubiquitous but can determine severe pneumonia [5], *Staphylococcus aureus* is part of the normal flora on human skin but may cause heart valve and bone infections [6], and *Achromobacter xylosoxidans* commonly present in soil and water might cause respiratory and urinary tract infections [7]. Recent research is now suggesting the existence of a two-way association between microbiota and the variability of many human traits. Indeed, dysbiosis—defined as an imbalance in microbiota profiles—has been associated with a range of complex diseases, including metabolic, immune, neurodegenerative, and neurological diseases [8,9,10,11]. Nonetheless, the underlying mechanisms of the reciprocal influence among individual or communities of microorganisms and human health are puzzling and need to be further clarified.

Molecular studies are now showing the complexity of microbial communities with a novel level of detail that allows scientists to combine the information from different biological layers (omics sciences) captured using newly available technologies. High-throughput sequencing approaches enable researchers to dissect the complexity of microbiota composition and metabolic potential in different anatomical niches [12]. Nonetheless, methodological and analytical hurdles still remain, including sample collection and storage strategies, choosing the most suitable molecular method, taxonomical classification at different ranks, metabolic pathway prediction, and host–pathogen interaction identification. 

In the current Special Issue entitled “The Interplay between Microbiota and Human Complex Traits”, we encourage the exploration of the relationship between microbiota and human complex traits and/or diseases, preventive and therapeutic measures to predict and contrast the progression of disease, as well as the investigation of novel methods to elucidate the underlying host–microbe connections.
